# Simulation-Based Training of Non-Technical Skills in Colonoscopy: Protocol for a Randomized Controlled Trial

**DOI:** 10.2196/resprot.7690

**Published:** 2017-08-04

**Authors:** Rishad Khan, Michael A Scaffidi, Catharine M Walsh, Peter Lin, Ahmed Al-Mazroui, Barinder Chana, Ruben Kalaichandran, Woojin Lee, Teodor P Grantcharov, Samir C Grover

**Affiliations:** ^1^ St. Michael's Hospital Division of Gastroenterology University of Toronto Toronto, ON Canada; ^2^ Hospital for Sick Children Division of Gastroenterology, Hepatology, and Nutrition, Learning Institute, and Research Institute University of Toronto Toronto, ON Canada; ^3^ The Wilson Centre University of Toronto Toronto, ON Canada; ^4^ St. Michael's Hospital Department of General Surgery University of Toronto Toronto, ON Canada

**Keywords:** simulation, colonoscopy, non-technical skills, skill acquisition

## Abstract

**Background:**

Non-technical skills (NTS), such as communication and professionalism, contribute to the safe and effective completion of procedures. NTS training has previously been shown to improve surgical performance. Moreover, increases in NTS have been associated with improved clinical endoscopic performance. Despite this evidence, NTS training has not been tested as an intervention in endoscopy.

**Objective:**

The aim of this study is to evaluate the effectiveness of a simulation-based training (SBT) curriculum of NTS on novice endoscopists’ performance of clinical colonoscopy.

**Methods:**

Novice endoscopists were randomized to 2 groups. The control group received 4 hours of interactive didactic sessions on colonoscopy theory and 6 hours of SBT. Hours 5 and 6 of the SBT were integrated scenarios, wherein participants interacted with a standardized patient and nurse, while performing a colonoscopy on the virtual reality (VR) simulator. The NTS (intervention) group received the same teaching sessions but the last hour was focused on NTS teaching. The NTS group also reviewed a checklist of tasks relevant to NTS concepts prior to each integrated scenario case and was provided with dedicated feedback on their NTS performance during the integrated scenario practice. All participants were assessed at baseline, immediately after training, and 4 to 6 weeks post-training. The primary outcome measure is colonoscopy-specific performance in the clinical setting.

**Results:**

In total, 42 novice endoscopists completed the study. Data collection and analysis is ongoing. We anticipate completion of all assessments by August 2017. Data analysis, manuscript writing, and subsequent submission for publication is expected to be completed by December 2017.

**Conclusions:**

Results from this study may inform the implementation of NTS training into postgraduate gastrointestinal curricula. NTS curricula may improve attitudes towards patient safety and self-reflection among trainees. Moreover, enhanced NTS may lead to superior clinical performance and outcomes in colonoscopy.

**Trial Registration:**

Clinicaltrial.gov NCT02877420; https://www.clinicaltrials.gov/ct2/show/NCT02877420 (Archived by WebCite at http://www.webcitation.org/6rw94ubXX NCT02877420)

## Introduction

Simulation-based training (SBT) in gastrointestinal (GI) endoscopy improves clinical performance among trainees [1-3]. However, the components of optimal simulation curricula are unclear. In procedural settings like surgery, the teaching of non-technical skills (NTS) has been shown to improve novice surgeons’ performance [4]. Despite this evidence, the impact of NTS training on endoscopy performance has not been explored.

NTS, such as teamwork, communication, situational awareness, and decision making, are important factors in healthcare regarding adverse health outcomes. A recent systematic review of critical incidents in intensive care units found that failures in non-technical domains contributed to a large proportion of medical errors [5]. Another systematic review found that deficiencies in NTS were associated with decreased technical skill in surgical settings [6].

Given the importance of these skills, several interventions have been proposed to enhance NTS among physicians. First, didactic training has been shown to improve attitudes and awareness of NTS. In a study among surgical residents, a curriculum featuring didactic teaching of NTS led to improved non-technical specific performance in the operating room, compared to conventional training (ie, daily activities on surgical wards, call schedules, and designated operating room time) [4]. Second, checklists have been shown to improve NTS, such as promoting adherence to procedural protocols, especially during surgical crises [7]. Finally, debriefing and feedback by expert instructors can allow trainees to acquire and consolidate relevant NTS. Among residents, both oral and videotape-assisted feedback can yield superior NTS acquisition compared to no feedback [8].

In endoscopy, improvement of NTS in the simulated setting is associated with superior colonoscopy skills [3]. However, the direct impact of NTS-specific training on clinical performance is unknown. This study aims to evaluate the impact of NTS training on clinical colonoscopy performance among novice endoscopists.

## Methods

**Figure 1:**
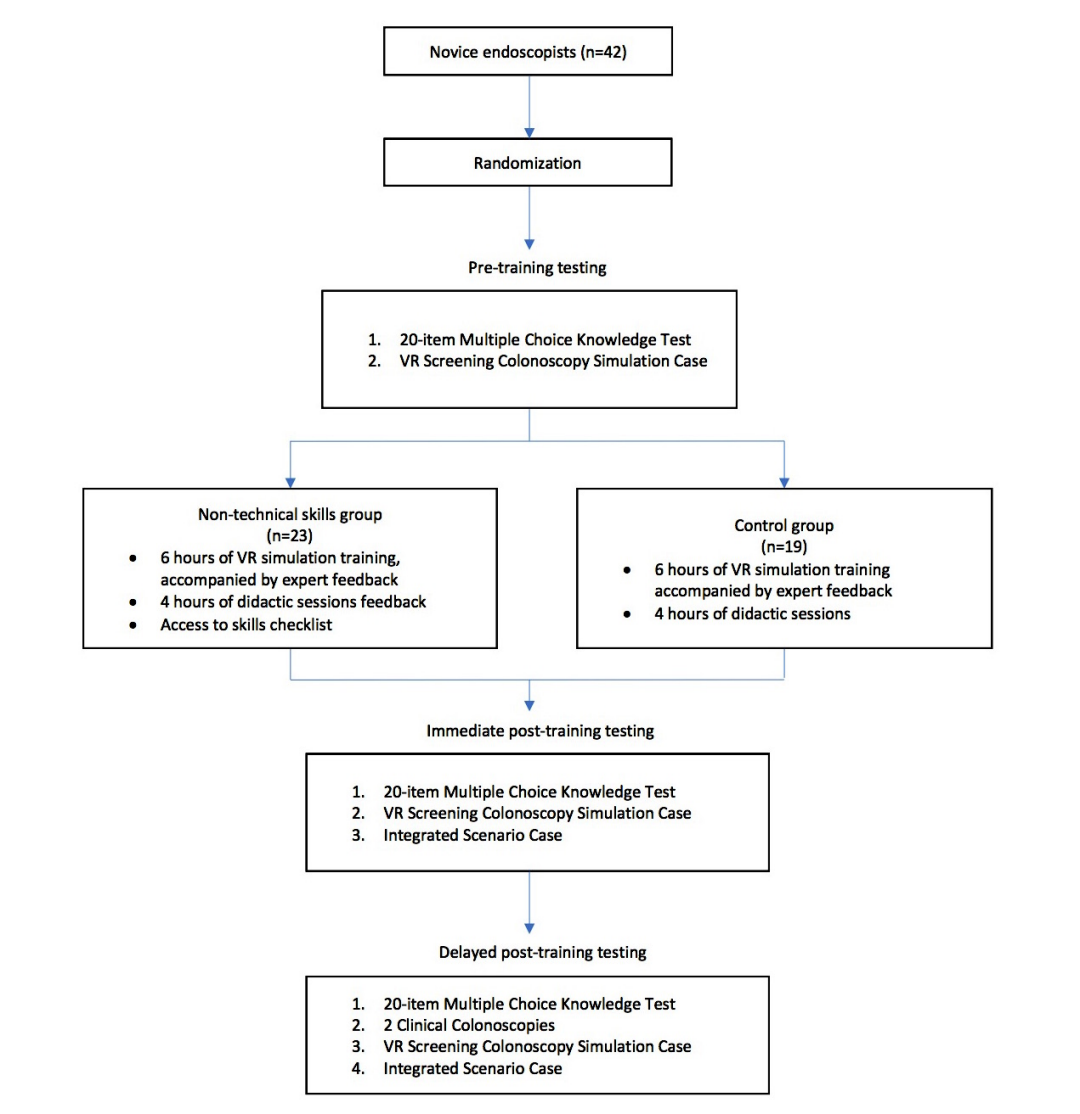
Study design.

This single-blind, parallel group, randomized controlled trial (RCT) is being conducted at a tertiary-care academic center. Research ethics approval was granted by the St. Michael’s Hospital Research Ethics Board (15-164). Recruitment for the study is complete. All testing and training took place at St. Michael’s Hospital (30 Bond Street, Toronto, Ontario, M5B 1W8). Informed written consent was acquired from all participants and patients involved in the study. The study design is summarized in Figure 1.

### Participants

A total of 42 postgraduate trainees enrolled in general surgery, adult gastroenterology, and internal medicine programs at the University of Toronto were recruited through purposive sampling. Participants were identified from a list of trainees rotating through the gastroenterology service at St. Michael’s Hospital and emailed with recruitment details. Study enrollment took place from June 2015 to June 2016. Participants were excluded if they performed 25 or more real or simulated endoscopic procedures at the time of their participation in the study.

### Simulation Devices

#### Bench Top, Low-Fidelity Simulator

The low-fidelity simulator is a validated bench-top endoscopy simulator that helps develop general endoscopic skills [9]. The simulator is comprised of a series of vertical wooden barriers with numbered holes conforming to 27 different sequences of varying complexity. An Olympus PCF-180 pediatric videocolonoscope (Olympus Canada) is used to navigate the defined sequences as quickly and accurately as possible, with visual output being displayed on a video monitor.

#### Virtual Reality, High-Fidelity Simulator

The high-fidelity simulator is the EndoVR endoscopy simulator (CAE Healthcare Canada). It models navigation through a colon, using a specialized endoscope that is inserted into a computer-based module with a screen showing the colonic lumen of a virtual patient. It provides both visual and haptic feedback related to the procedure. The VR simulator has several standardized case-based scenarios of varying complexity for colonoscopy.

### Experimental Design

#### Baseline Questionnaire

A written questionnaire is administered to all participants at the start of the training to collect demographic and background information including age, sex, level of training, previous endoscopy experience, and nature of experience (if applicable). Previous experience with team sports and video games is also assessed, as these activities correlate with NTS and baseline endoscopic skill, respectively (Multimedia Appendix 1) [10].

#### Pre-Test

Participants take part in a pre-test designed to assess their baseline (1) knowledge of colonoscopy (knowledge test); (2) technical skills (VR simulation test); and (3) NTS (VR simulation-based “integrated scenario” test). No feedback is provided at any point during the pre-test.

The knowledge test is a 30-minute test containing 20 multiple choice questions (MCQ) designed to assess participants’ theoretical knowledge of colonoscopy, including indications, sedation, safety, findings, pathology, and follow-up (Multimedia Appendix 2).

Participants' baseline endoscopic technical proficiency is assessed through the completion of a colonoscopy procedure on the VR simulator (EndoVR Colonoscopy Module 3). This scenario simulates a screening colonoscopy, without the need for any type of intervention, such as biopsy. There is a time limit of 30 minutes per procedure. An expert rater assesses performance, but does not provide assistance. Participants are videotaped to obtain performance measures with their faces hidden to ensure anonymity. Prior to starting the procedure, participants complete a questionnaire to measure their self-efficacy.

Following the simulator-only test, participants complete an integrated scenario format test to assess their baseline endoscopic non-technical proficiency. The integrated scenario requires participants to perform a colonoscopy procedure on the VR simulator while interacting with an endoscopic nurse and a standardized patient (SP) [11]. The simulated procedure mimics the setup of an endoscopic suite with the VR simulator positioned next to a patient table. An SP, who receives instructions regarding their medical role, acts out a scenario on colon cancer screening. Trainees are expected to explain the colonoscopy procedure, its benefits and risks, and obtain procedural consent. Trainees then carry out the procedure on the VR simulator (EndoVR Polypectomy Module #3) while responding to the patient and interacting with the standardized nurse (SN), as appropriate. The SP acts out cues from the VR simulator when the simulator signals that the procedure has exceeded its threshold for discomfort. The performances of all participants are videotaped in a similar manner to the VR simulation test to obtain performance measures. Participants are given a maximum of 45 minutes to complete the procedure. Prior to starting the procedure, participants complete a questionnaire to measure their self-efficacy.

#### Training Intervention

Participants are subsequently randomized using a randomization algorithm to 1 of 2 groups (control and intervention), following a 1:1 allocation distribution.

The control group receives 4 hours of interactive, small-group, didactic and hands-on sessions on the theory of colonoscopy, led by an expert academic gastroenterologist. The core curriculum was designed based on the American Society for Gastrointestinal Endoscopy (ASGE) colonoscopy curriculum and a detailed endoscopic training textbook and includes teaching on anatomy, pathophysiology, indications, risks and benefits of the procedures, training on specific elements of performance of colonoscopy procedures (eg, one-handed steering), and strategies for loop reduction, terminal ileal cannulation, and adequate visualization of mucosa [12,13]. This curriculum has been shown to be effective when compared to self-regulated learning on the simulator [3]. The sessions are interlaced with 6 hours of expert-assisted instruction on both the low-fidelity simulator (1 hour) and on the high-fidelity VR simulator (5 hours). Six modules of increasing difficulty in colonoscopy and colonoscopy polypectomy are taught with one-on-one feedback from an expert academic endoscopist. The endoscopy instructor demonstrates techniques, answers questions, and provides individualized performance feedback on global performance. The final 2 hours spent on the high-fidelity scenario use the integrated scenario, which features an SP and SN.

The intervention (NTS) group receives the same first 3 hours of interactive, small-group, didactic and hands-on sessions as the control group. The 4th hour of didactic sessions focuses on NTS, which includes a discussion of the major areas of NTS. Participants also watch a video demonstrating an ideal endoscopic procedure (ie, benchmark video) in terms of technical and NTS. These sessions and the video introduce trainees to the Endoscopic Non-Technical Skills (E-NTS) Checklist, which is provided for them to use during the integrated scenario training (Multimedia Appendix 3). This checklist was developed per evidence-based recommendations and targets NTS in endoscopy [14]. The NTS group also receives 6 hours of expert-assisted instruction on both the low-fidelity simulator (1 hour) and on the high-fidelity VR simulator (5 hours). Six modules of increasing difficulty in colonoscopy and colonoscopy polypectomy are taught with one-on-one feedback from an expert academic endoscopist. The endoscopy instructor demonstrates techniques, answers questions, and provides individualized performance feedback on global performance with a focus on NTS. Similar to the control group, the final 2 hours spent on the high-fidelity scenario use the integrated scenario. Terminal feedback dedicated to NTS is given after each integrated scenario by the instructor. Finally, participants in this group have access to the E-NTS Checklist during training in the integrated scenario, as participants can view the checklist prior to and after each case.

#### Post-Test

A post-test is administered after completion of the training period to compare skill and knowledge acquisition between the control and intervention groups. The immediate post-test is designed to evaluate trainees’ (1) knowledge acquisition (knowledge test); (2) technical skills acquisition (VR simulation test); and (3) NTS acquisition (VR simulation-based integrated scenario test). No feedback is provided during the post-test.

Participants’ knowledge acquisition is evaluated using the same MCQ test conducted at baseline. Again, trainees have 30 minutes to complete the 20 questions.

Participants' technical skill acquisition is assessed through the completion of the same colonoscopy procedure on the VR simulator (EndoVR Colonoscopy Module 3). The time limit is 30 minutes. Prior to starting the procedure, participants complete a questionnaire to measure their self-efficacy.

Participants' NTS acquisition is assessed using the integrated scenario procedure on the VR simulator (EndoVR Polypectomy Module #3), while also responding to the patient and interacting with the nurse, as appropriate. The time limit is 45 minutes. Prior to starting the procedure, participants complete a questionnaire to measure their self-efficacy.

#### Delayed Testing

A delayed-test is administered 4 to 6 weeks after completion of the training period to compare retention and transfer of skill between the control and intervention groups. It consists of (1) a knowledge test; (2) a VR simulation test; (3) a VR simulation-based integrated scenario test; and (4) a patient-based transfer test.

Participants’ knowledge acquisition is evaluated using the same MCQ test conducted at baseline and during the post-test. Trainees have 30 minutes to complete 20 questions.

Participants' technical skill acquisition is assessed through the completion of the same colonoscopy procedure on the VR simulator (EndoVR Colonoscopy Module 3). The time limit is 30 minutes. Prior to starting the procedure, participants complete a questionnaire to measure their self-efficacy.

Participants' NTS acquisition is assessed using the integrated scenario procedure on the VR simulator (EndoVR Polypectomy Module #3), while also responding to the patient and interacting with the nurse, as appropriate. The time limit is 45 minutes. Prior to starting the procedure, participants complete a questionnaire to measure their self-efficacy.

Participants’ transfer of skills to the clinical setting is assessed using live colonoscopies. Each participant completes 2 colonoscopies on real patients 4 to 6 weeks after the training period. These procedures are videotaped in a manner that anonymizes the participant and patient. Procedures on patients with a history of colonic or pelvic surgery or difficult colonoscopy are excluded. Sedation and monitoring are carried out per standard practice at the endoscopy unit. An experienced attending endoscopist (greater than 1000 completed procedures) provides verbal and/or hands-on assistance as necessary and takes over if the participant cannot complete the procedure or if any concerns regarding patient safety arise.

### Outcome Measures

The primary outcome measure is the difference in performance between the control and intervention groups during participants’ 2 clinical colonoscopies. Each videotaped clinical colonoscopy will be independently assessed by 2 experienced endoscopists using the Joint Advisory Group for GI Endoscopy Direction Observation of Procedural Skills (JAG DOPS) (Multimedia Appendix 4) [15]. The raters will be blinded to the group assignment. Training on how to use the tool will be provided for raters by the investigators of the study.

Secondary outcome measures include the differences between the control and intervention groups with respect to (1) procedural knowledge, as assessed by the knowledge MCQ tests; (2) NTS performance during the clinical colonoscopies, as assessed by the Modified Objective Structured Assessment of Non-Technical Skills (M-OSANTS) for colonoscopy, which has been previously validated for surgery and modified for endoscopy [4] (Multimedia Appendix 5); (3) clinical performance on clinical colonoscopies, as assessed by the Gastrointestinal Endoscopy Competency Assessment Tool (GiECAT) [16] (Multimedia Appendix 6); (4) technical performance on a VR simulated colonoscopy after training and 4 to 6 weeks after training (immediate and delayed post-training assessments, respectively), as assessed by the JAG DOPS and the GiECAT; (5) technical and non-technical performance during an integrated scenario format test 4 to 6 weeks after training, as assessed by the JAG DOPS, GiECAT, and M-OSANTS; (6) patient comfort during the clinical colonoscopies, as assessed by the Nurse-Assessed Patient Comfort Score (NAPCOMS) [17] (Multimedia Appendix 7); (7) participant self-efficacy, as measured by an adapted scale based on the General Self-Efficacy Scale (GSE) [18,19] (Multimedia Appendix 8); and (8) global performance and communication skills during integrated scenarios as assessed by the Integrated Scenario Global Rating Form (ISGRF) and Integrated Scenario Communication Rating Form (ISCRF) [11,20,21], respectively (Multimedia Appendices 9 and ).

Experienced endoscopists will assess participants’ colonoscopy-specific skills, technical skills, and NTS during the pre-training, immediate, and delayed post-training simulation-based assessments.

### Analysis Plan

Statistical analyses will be performed using SPSS version 20 (SPSS, Inc.). All statistical tests will be considered significant at *P* less than .05.

### Baseline Questionnaire

Patient demographics and baseline variables will be characterized with descriptive statistics, using mean with standard deviation and number with frequency for continuous and categorical variables, respectively.

### Clinical Performance

Clinical performance during the live colonoscopies for each group will be determined by comparing the scores from the DOPS, GiECAT, NAPCOMS, and Modified-OSANTS. Specifically, a mixed factor 2 (control curriculum versus intervention curriculum) times 2 (procedure 1 versus procedure 2) analysis of variance (ANOVA) will be used to determine whether there is a difference based on the rating scales. The Tukey honest significant difference (HSD) test will be used as a post-hoc analysis to determine any significant differences.

#### Technical Performance

Technical performance on the simulator for each group will be determined by comparing the scores from the DOPS and GiECAT. Specifically, a mixed factor 2 (control curriculum versus intervention curriculum) times 3 (pre-test, post-test, delayed test) ANOVA will be used to determine whether there is a difference based on the rating scales. The Tukey HSD test will be used as a post-hoc analysis to determine any significant differences.

#### Non-Technical Performance

Non-technical performance on the simulator for each group will be determined by comparing the scores from the M-OSANTS, ISGRF, and ISCRF. Specifically, a mixed factor 2 (control curriculum versus intervention curriculum) times 3 (pre-test, post-test, delayed test) ANOVA will be used to determine whether there is a difference based on the rating scales. The Tukey HSD test will be used as a post-hoc analysis to determine any significant differences.

### Self-Efficacy

Self-efficacy during the simulated setting for each group will be determined by comparing the scores from the GSE. Specifically, a mixed factor 2 (control curriculum versus intervention curriculum) times 3 (pre-test, post-test, delayed test) ANOVA will be used to determine whether there is a difference based on the rating scales. The Tukey HSD test will be used as a post-hoc analysis to determine any significant differences.

### Sample Size Estimation

A power analysis was computed using G*Power version 3.1.9 [22]. Using a previous study that evaluated an NTS training curriculum in surgery, we conducted the analysis using the relevant effect size [4]. Based on an effect size of f=0.65, an alpha of .05 (2-tailed), a beta of .20, 2 groups, and 3 measurements, 16 participants are required to achieve a power of greater than 0.80 using repeated measures ANOVA (between-factors). Furthermore, a previous study comparing a curriculum in endoscopic simulation found that a minimum of 15 participants per group was sufficient to detect a significant difference [3]. To accommodate for a projected 20% dropout and/or non-response, we recruited a total of 42 participants.

## Results

A total of 42 participants were recruited, randomized, and completed the study. No participants were lost to follow-up. Through a 1:1 allocation distribution, 19 individuals were randomized into the control group and 23 individuals were randomized into the NTS group. Their demographic information and endoscopic experiences are summarized in Table 1. Two experienced endoscopists were recruited to assess participants’ videotaped performances and we anticipate completion of all assessments by August 2017. Data analysis, manuscript writing, and subsequent submission for publication is expected to be completed by December 2017.

**Table 1:** Baseline demographic characteristics and endoscopy experience of participants (N=42).

Variable		Control group (n=19)	Intervention (NTS^a^) group (n=23)
Age in years, mean (SD)		29.5 (1.8)	28.1 (2.1)
**Gender, n (%)**			
	Male	11 (58)	16 (70)
	Female	8 (42)	7 (30)
**Training program, n (%)**			
	Gastroenterology	4 (21)	6 (26)
	General surgery	13 (68)	16 (70)
	Internal medicine	2 (11)	1 (4)
**Previous endoscopic experience, mean (SD)**			
	Number of independent colonoscopies	0.11 (0.46)	0.30 (0.82)
	Number of assisted colonoscopies	2.63 (4.66)	1.70 (2.50)

^a^NTS: non-technical skills.

## Discussion

### Principal Findings

The practice of effective NTS is critical in procedural medicine. In addition, deficiencies in NTS are associated with adverse patient outcomes [5,6]. However, there is evidence that NTS training can improve surgical performance in the simulated setting, lead to better attitudes towards patient safety, and promote more self-reflection among trainees [4,23,24].

Previous studies have explored NTS in GI endoscopy. Matharoo et al shared their perspectives on implementing an endoscopy safety checklist to decrease adverse health outcomes [25]. This paper reported on the implications, logistics, and uptake of a safety checklist, and did not present any outcome data. A 2014 report implementing an NTS curriculum found that it improved patient safety knowledge and attitudes among multi-disciplinary endoscopy teams [24]. In another study, an RCT comparing 2 curricula of SBT in endoscopy found that improved NTS in the simulated setting were associated with superior clinical performance [3]. However, that study did not test NTS training as an intervention.

### Conclusion

This study aims to evaluate the direct impact of NTS training on clinical colonoscopy performance. Results can inform the potential implementation of NTS into postgraduate gastrointestinal curricula.

## Appendix

Multimedia Appendix 1 Baseline demographic and endoscopic experience.

Multimedia Appendix 2 Knowledge test questions.

Multimedia Appendix 3 Endoscopic non-technical skills (E-NTS) checklist.

Multimedia Appendix 4 Joint Advisory Group on GI Endoscopy Direct Observation of Procedural Skills (JAG DOPS) form.

Multimedia Appendix 5 Modified Objective Structured Assessment of Non-Technical Skills (M-OSANTS) form.

Multimedia Appendix 6 Gastrointestinal Endoscopy Competency Assessment Tool (GiECAT) form.

Multimedia Appendix 7 Nurse Assessed Patient Comfort Score (NAPCOMS) form.

Multimedia Appendix 8 General self-efficacy assessment.

Multimedia Appendix 9 Integrated Scenario Global Rating Form (ISGRF).

Multimedia Appendix 10 Integrated Scenario Communication Rating Form (ISCRF).

## Abbreviations

ANOVA: analysis of variance
E-NTS: endoscopic non-technical skills
GI: gastrointestinal
GiECAT: Gastrointestinal Endoscopy Competency Assessment Tool
GSE: General Self-Efficacy Scale
HSD: honest significant difference
ISCRF: Integrated Scenario Communication Rating Form
ISGRF: Integrated Scenario Global Rating Form (ISGRF)
JAG DOPS: Joint Advisory Group for GI Endoscopy Direction Observation of Procedural Skills
M-OSANTS: Modified Objective Structured Assessment of Nontechnical Skills
MCQ: multiple choice questions
NAPCOMS: Nurse-Assessed Patient Comfort Score
NTS: non-technical skills
RCT: randomized controlled trial
SBT: simulation-based training
SN: standardized nurse
SP: standardized patient
VR: virtual reality
