# Supplementary material for: Simulation-Based Training of Non-Technical Skills in Colonoscopy: Protocol for a Randomized Controlled Trial
Source: JMIR Res Protoc. 2017 Aug 4;6(8):e153. doi: 10.2196/resprot.7690 (PMC5562936; doi:10.2196/resprot.7690)
Supplement: Multimedia Appendix 1 [file resprot_v6i8e153_app1.pdf]

APPENDIX I:

BASELINE QUESTIONNAIRE

Participant ID Number: ___________________

1) Sex: □ Female □ Male

2) Age: ______________

3) Handedness: □ Right □ Left □ Ambidextrous

4) Year of graduation from Medical School: ______________

5) Programme:

□ Adult Gastroenterology
□ Pediatric Gastroenterology
□ General Surgery
□ Other (please specify: _________________________________)

6) Level of training:

□ PGY 1
□ PGY 2
□ PGY 3
□ PGY 4
□ PGY 5
□ Other (please specify: _________________________________)

7) Do you have previous experience in playing video games? □ Yes □ No

If yes, please specify:

(a) How many hours do you play on average per week? __________
(b) What types of games do you play? □ Sports □ Role-playing □ Real-time strategy □ Other (please describe)

8) Do you have previous experience in performing gastrointestinal endoscopy in the clinical or simulated setting? □ Yes □ No

If yes, please specify:

(c) Number of previous upper endoscopies in the ***clinical*** setting (attempted or completed): __________
(d) Number of previous upper endoscopies in the ***simulated*** setting (attempted or completed): ________
(e) Number of previous colonoscopies in the ***clinical*** setting (attempted or completed): _____________
(f) Number of previous colonoscopies in the ***simulated*** setting (attempted or completed): ___________
(g) Number of previous sigmoidoscopies in the ***clinical*** setting (attempted or completed): ___________
(h) Number of previous sigmoidoscopies in the ***simulated*** setting (attempted or completed): _________
(i) Number of other ***clinical*** GI endoscopy procedures (please specify procedure): _________________

____

(j) Number of other ***simulated*** GI endoscopy procedures (please specify procedure): ________________

____
