# Supplementary material for: Simulation-Based Training of Non-Technical Skills in Colonoscopy: Protocol for a Randomized Controlled Trial
Source: JMIR Res Protoc. 2017 Aug 4;6(8):e153. doi: 10.2196/resprot.7690 (PMC5562936; doi:10.2196/resprot.7690)
Supplement: Multimedia Appendix 2 [file resprot_v6i8e153_app2.pdf]

Endoscopy Knowledge Post-Test

This is the knowledge test for the University of Toronto endoscopic simulation course. Please type in your "endo" number to begin the test. The test consists of 17 questions for a total of 20 points.

The knowledge questions are on the next two pages. You have 30 minutes to answer these questions.

These answers are confidential, and your responses and score are only for the purposes of our research studies. They will not be used as part of your residency training assessments, or as part of competency assessments.

*Required

1. **Please type in your "endo" number login to start** *
   ie, "Endo10"

   ____

Endoscopic Knowledge Test (page 1 of 2)

2. **Question 1 (1 point)**
   Name the endoscopic device depicted below.
   Mark only one oval.

   - ⬭ A. Endoloop
   - ⬭ B. Endoscopic snare
   - ⬭ C. Endoscopic biopsy forceps
   - ⬭ D. Gold probe
   - ⬭ E. Oval probe

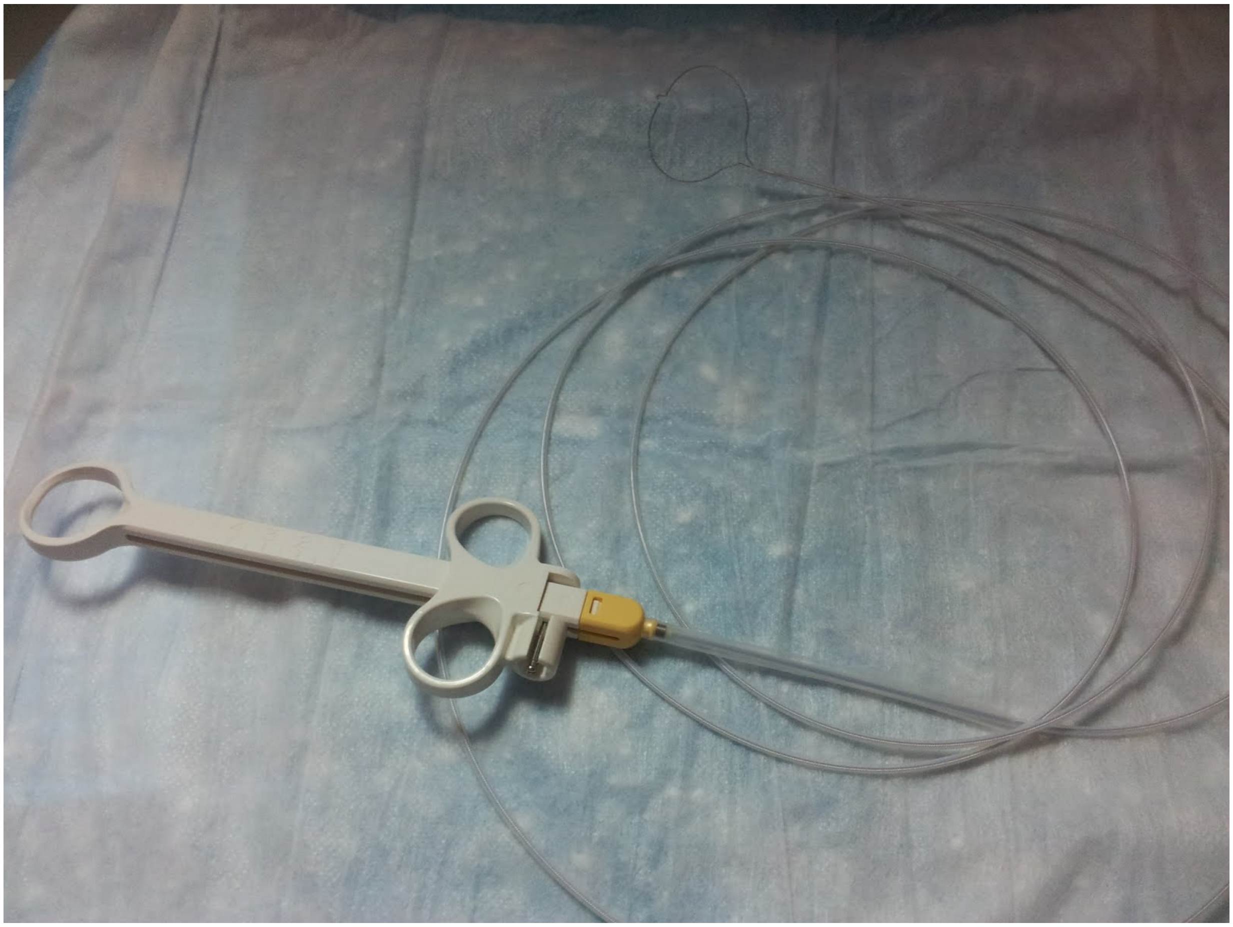

3. **Question 2 (1 point)**
What type of sigmoid colon loop is most beneficial for entry into the descending colon?
Mark only one oval.

- A. Alpha-loop
- B. Reverse alpha-loop
- C. Gamma-loop
- D. N-loop
- E. Reverse-N loop

4. **Question 3 (1 point)**
Below is an endoscopic image of the larynx taken with a gastroscope. Which location is representative of the ideal place to position the gastroscope to intubate the esophagus?
Mark only one oval.

- A
- B
- C
- D

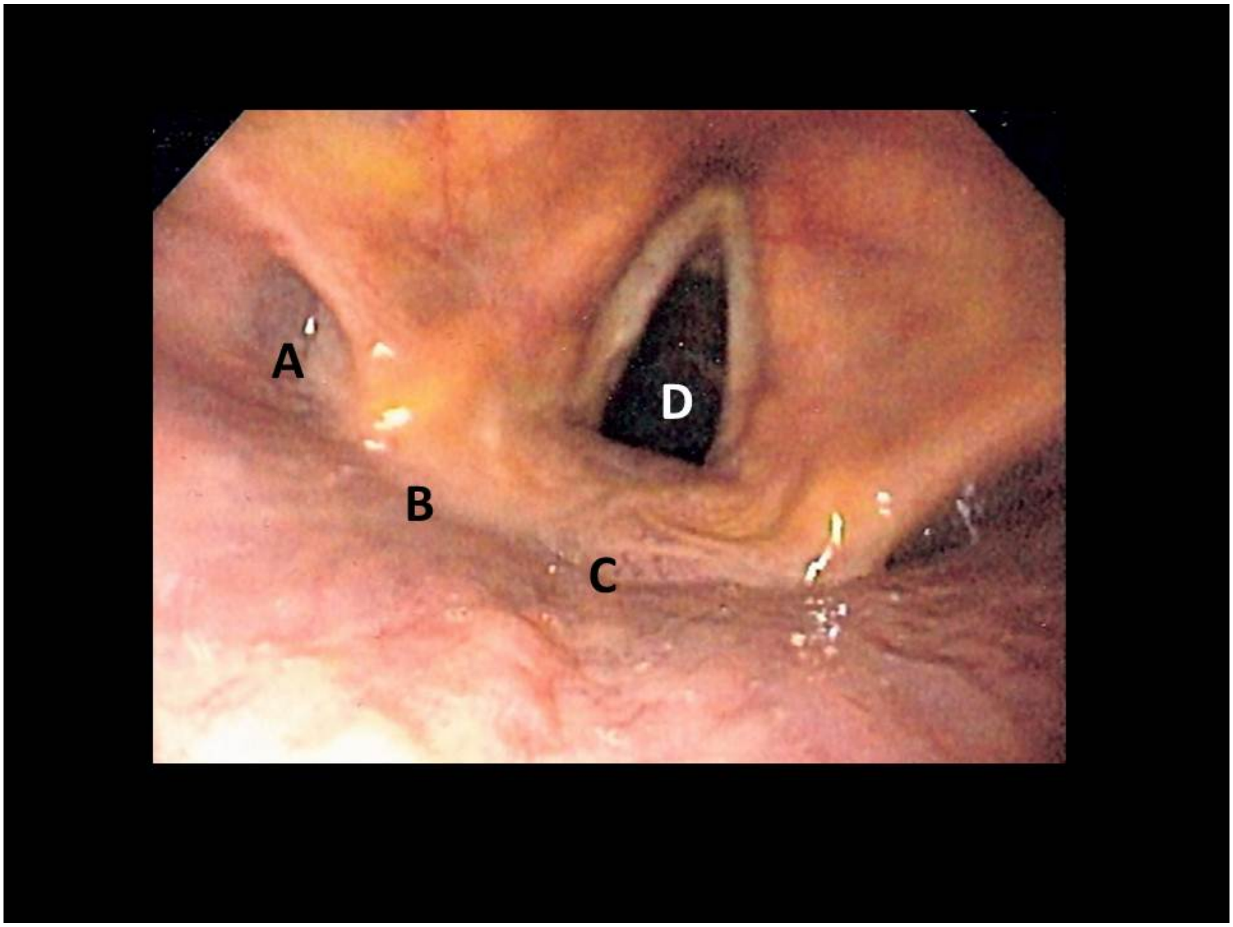

5. **Question 4 (1 point)**

Which of the images below is an endoscopic image of the cecum?

Mark only one oval.

- Image A
- Image B
- Image C
- Image D

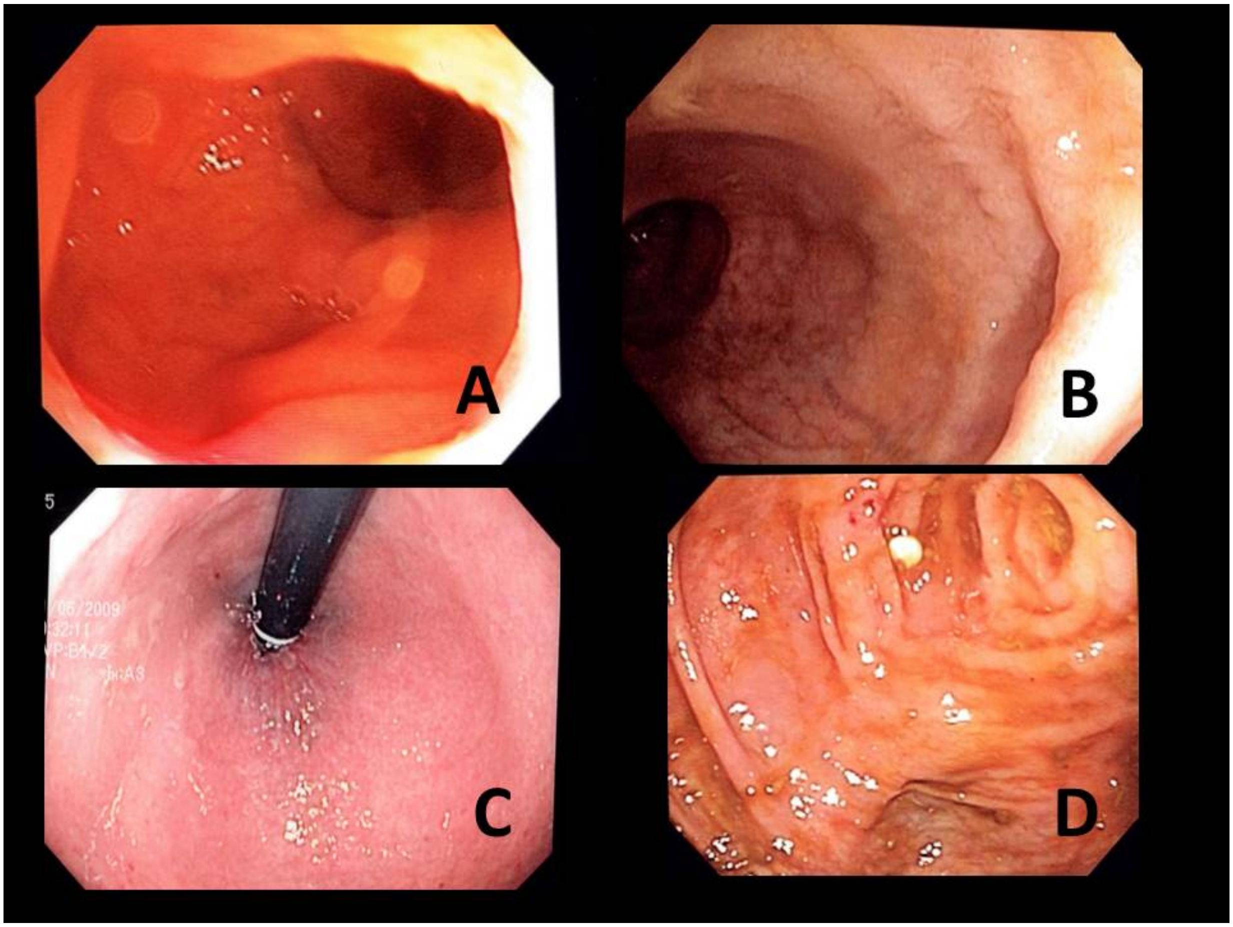

6. **Question 5 (1 point)**
The pit pattern of polyps is commonly used as an endoscopic classification to determine the likely histopathology. What is the name of the classification most commonly used for pit patterns?
Mark only one oval.

- A. Kudo classification
- B. Maclean classification
- C. Yoshida classification
- D. Haggitt classification
- E. Sarin classification

7. **Question 6 (1 point)**
The pit patterns of the polyp depicted below have been enhanced by use of ambient light of blue-green wavelength (approximately 450 - 540 nm). What is the name of this technology?
Mark only one oval.

- A. Confocal microscopy
- B. Optical coherence tomography
- C. Chromoendoscopy
- D. FICE (Fuji intelligent chromoendoscopy)
- E. Narrow band imaging

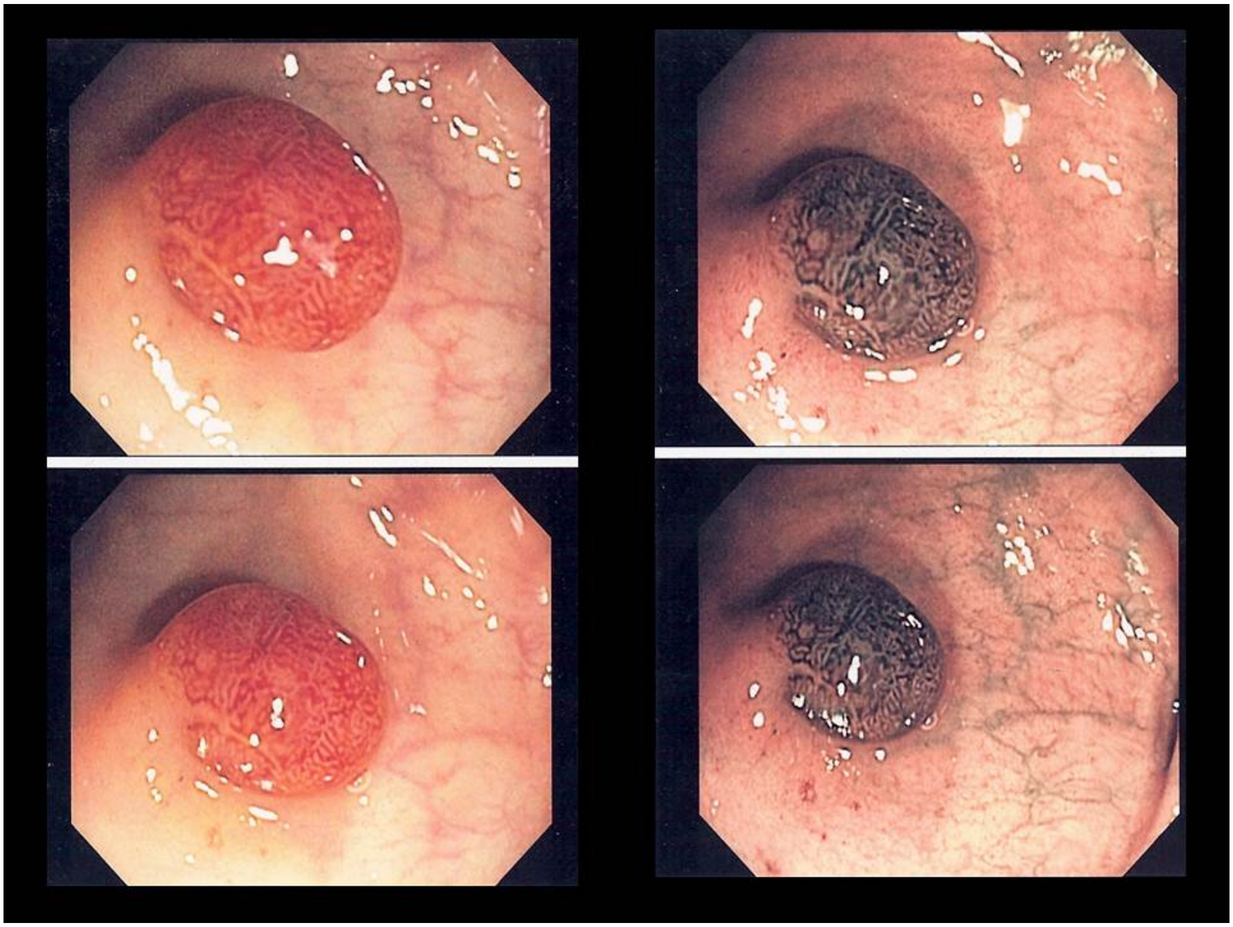

8. **Question 7 (1 point)**
With a well prepared colonoscopy to the cecum performed by an experienced endoscopist, what is the approximate risk of missed advanced neoplasia?
Mark only one oval.

- A. 1%
- B. 3-5%
- C. 5-10%
- D. >10%

9. **Question 8 (1 point)**
Below is an endoscopic view of a patient's esophagus. What is the endoscopic diagnosis?
Mark only one oval.

- A. Eosinophilic esophagitis
- B. Radiation esophagitis
- C. Mosaic esophagus
- D. Barrett's esophagus
- E. Diffuse-type squamous cell carcinoma

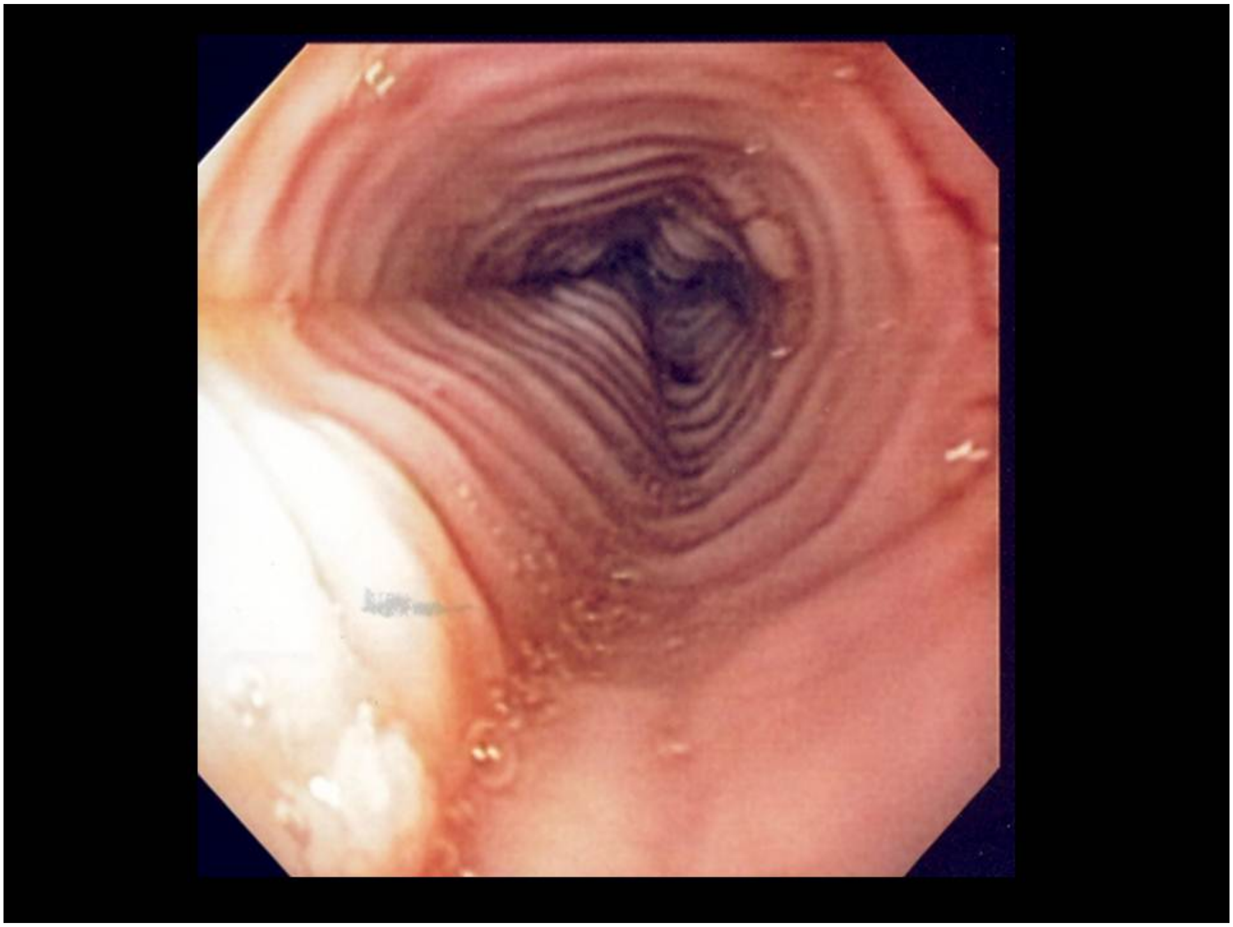

Endoscopy knowledge test (page 2 of 2)

10. **Question 9 (1 point)**
Which of the following is a commonly used bowel preparation score for colonoscopy?
Mark only one oval.

- A. Los Angeles bowel preparation score
- B. Ottawa bowel preparation score
- C. King's College bowel preparation score
- D. Chicago bowel preparation score
- E. Washington bowel preparation score

Question 10 (4 points)

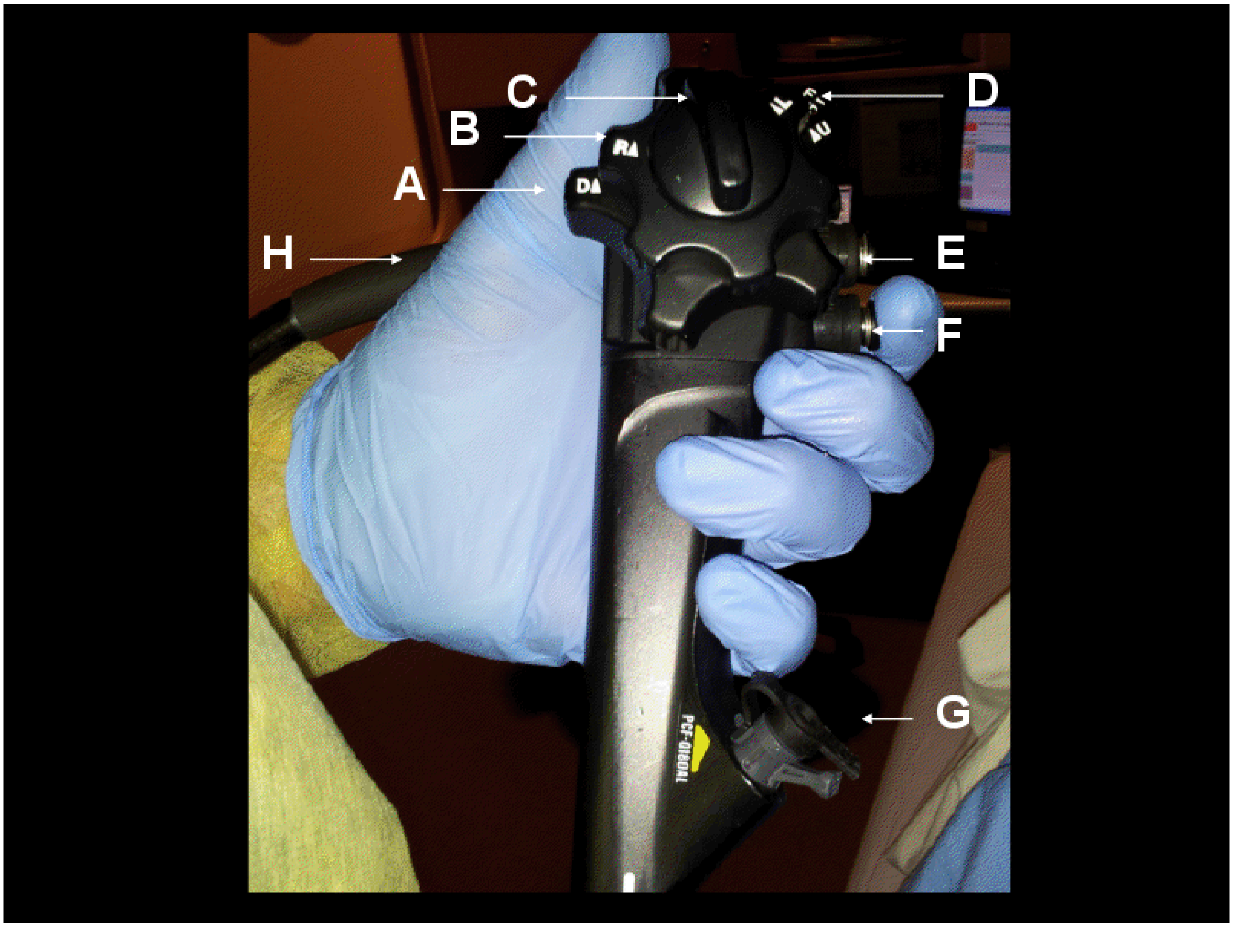

Identify the labelled parts of the colonoscope head (4 points; 0.5 points each)

11. **A**

12. **B**

13. **C**

14. **D**

15. **E**

16. **F**

17. **G**

18. **H**

Question 11 (1 point)

Certain skills necessary for endoscopic performance may be independent of the technical performance of the procedure. Name four non-technical skills that you would view as important in the performance of endoscopic procedures.

19. **A**

20. **B**

21. **C**

22. **D**

23. **Question 12 (1 point)**
Which of the following is not a risk factor for colonic perforation at the time of colonoscopy
Mark only one oval.

- A. Barotrauma
- B. Mucosal injection
- C. Sigmoid looping
- D. Trainee endoscopist performing colonoscopy
- E. Resection of sessile polyp

24. **Question 13 (1 point)**
A 50 year old patient presents for a screening colonoscopy to your open access endoscopy unit. They have completed a 4L bowel preparation. Upon taking a history, you discover that they had a colonoscopy 2 years ago to work up anorectal bleeding but you don’t have the results. What is the appropriate course of action?
Mark only one oval.

- A. Complete a screening colonoscopy because the indication of colorectal cancer screening is different than anorectal bleeding.
- B. Another screening colonoscopy is not needed; send patient home.
- C. Make concerted effort to track down report of prior colonoscopy and if not obtained, then proceed with screening colonoscopy.
- D. Make concerted effort to track down report of prior colonoscopy and if not obtained, then explain to patient that they can choose to defer procedure until report is obtained and reviewed
- E. Your endoscopic skills exceed that of the previous endoscopist and the procedure must be repeated.

25. **Question 14 (1 point)**
A unilingual Azerbaijani woman comes in for a gastroscopy to work up epigastric pain. She is unable to understand the consent process, which of the following is most correct?
Mark only one oval.

- A. Do a physical examination; if she has abdominal discomfort, continue with gastroscopy.
- B. Ask the patient’s daughter to translate the consent form.
- C. Reschedule the gastroscopy and ask her to bring a translator.
- D. Reschedule the gastroscopy and arrange for a translator to accompany her.
- E. Obtain consent from daughter and proceed with procedure

26. **Question 15 (1 point)**
You are completing a screening colonoscopy and discover a 12 mm pedunculated polyp in the transverse colon. The endoscopy nurse suggests the use of a 10 mm snare and no electrocautery, but you believe a 15 mm snare with electrocautery would be more appropriate. What do you do?
Mark only one oval.

- A. Take the nurse’s suggestion since he/she is more experienced than yourself.
- B. Use a 15 mm snare and ignore the nurse.
- C. Ask the nurse for the rationale for the smaller snare and the absence of cautery and verbalize your decision to proceed with the 15 mm snare with cautery.
- D. Discuss with the nurse why you wish to proceed with the larger snare and electrocautery before proceeding with the polypectomy.
- E. Call in a colleague to assist in the decision making.

27. **Question 16 (1 point)**

You are completing a polypectomy of a 2 cm Paris II-a (flat) polyp in the cecum of a 65 year old male. After the polypectomy you notice a perforation in the cecum. You aim to close the defect with endoclips, start antibiotics, and arrange imaging and possible surgery. Which of the following would not be appropriate?

Mark only one oval.

- A. Start the plan above for the management of the patient’s complication, as soon as possible.
- B. Inform family members of the procedure’s complications before proceeding with the above plan.
- C. Ensure airway, breathing and circulation are intact above all else.
- D. Call for extra assistance into the room as soon as possible.
- E. Stay calm as you manage the situation.

28. **Question 17 (1 point)**

You perform a screening colonoscopy on an anemic 50 year old male who complains of weight loss, altered bowel habits and blood in his stool. You identify the patient is suffering from colorectal cancer. Which one of the following would not be appropriate in explaining the results of the colonoscopy to the patient?

Mark only one oval.

- A. Ensure the results are delivered in a private setting
- B. Do not inform the patient of the suspected diagnosis until you have the pathology results
- C. Ensure to be empathetic during delivery of the results of the colonoscopy
- D. Establish that the patient understands the results after you explain it to them
- E. Be aware of the patient’s reaction and tone as you are delivering the news

TEAMSTEPPS TEAMWORK ATTITUDES QUESTIONNAIRE

With respect to how each question applies to interactions with a team in ENDOSCOPY, please respond to the questions below by placing a checkmark in the box that corresponds to your level of agreement from Strongly Disagree to Strongly Agree. We realize that the questions may be a little vague but please select only one response for each question.

Team Structure

29. **1. It is important to ask patients and their families for feedback regarding patient care before/in/after endoscopy** *

Mark only one oval.

- Strong disagree
- Disagree
- Neutral
- Agree
- Strongly agree

30. **2. Patients are a critical component of the care team in surgery and endoscopy** *

Mark only one oval.

- Strong disagree
- Disagree
- Neutral
- Agree
- Strongly agree

31. **3. The Department of Surgery or Division of Gastroenterology influences the success of direct care teams in surgery and endoscopy** *

Mark only one oval.

- Strong disagree
- Disagree
- Neutral
- Agree
- Strongly agree

32. **4. In surgery and endoscopy, the mission of the team is of greater value that the goals of individual team members** *

Mark only one oval.

- Strong disagree
- Disagree
- Neutral
- Agree
- Strongly agree

33. **5. Effective team members can anticipate the needs of other team members** *

Mark only one oval.

- Strong disagree
- Disagree
- Neutral
- Agree
- Strongly agree

34. **6. High-performing teams in health care share common characteristics with high-performing teams in other industries** *

Mark only one oval.

- Strong disagree
- Disagree
- Neutral
- Agree
- Strongly agree

Leadership

35. **7. It is important for a surgeon-leader or GI-leader to share information with team members** *
Mark only one oval.

- Strong disagree
- Disagree
- Neutral
- Agree
- Strongly agree

36. **8. Leaders should create informal opportunities for team members to share information** *
Mark only one oval.

- Strong disagree
- Disagree
- Neutral
- Agree
- Strongly agree

37. **9. Effective leaders view honest mistakes as meaningful learning opportunities** *
Mark only one oval.

- Strong disagree
- Disagree
- Neutral
- Agree
- Strongly agree

38. **10. It is a leader’s responsibility to model appropriate team behaviour** *
Mark only one oval.

- Strong disagree
- Disagree
- Neutral
- Agree
- Strongly agree

39. **11. It is important for surgeon-leaders or endoscopy-leaders to take time to discuss their team members' plans for each patient** *

Mark only one oval.

- Strong disagree
- Disagree
- Neutral
- Agree
- Strongly agree

40. **12. Team leaders should ensure that team members help each other out when necessary** *

Mark only one oval.

- Strong disagree
- Disagree
- Neutral
- Agree
- Strongly agree

Situation monitoring

41. **13. Individuals can be taught how to scan the patient environment in the OR or procedure room for important situation cues.** *

Mark only one oval.

- Strong disagree
- Disagree
- Neutral
- Agree
- Strongly agree

42. **14. Monitoring patients provides an important contribution to the effective performance of the team** *

Mark only one oval.

- Strong disagree
- Disagree
- Neutral
- Agree
- Strongly agree

43. **15. Even individuals who are not part of the direct care team should be encouraged to scan for and report changes in patient status** *

Mark only one oval.

- Strong disagree
- Disagree
- Neutral
- Agree
- Strongly agree

44. **16. It is important to monitor the emotional and physical status of other team members** *

Mark only one oval.

- Strong disagree
- Disagree
- Neutral
- Agree
- Strongly agree

45. **17. It is appropriate for one team member to offer assistance to another who may be too tired or stressed to perform a task** *

Mark only one oval.

- Strong disagree
- Disagree
- Neutral
- Agree
- Strongly agree

46. **18. Team members who monitor their emotional and physical status on the job are more effective** *

Mark only one oval.

- Strong disagree
- Disagree
- Neutral
- Agree
- Strongly agree

Mutual support

47. **19. To be effective, team members should understand the work of their fellow team members.** *

Mark only one oval.

- Strong disagree
- Disagree
- Neutral
- Agree
- Strongly agree

48. **20. Asking for assistance from a team member us a sign that that individual does not know how to do his/her job effectively** *

Mark only one oval.

- Strong disagree
- Disagree
- Neutral
- Agree
- Strongly agree

49. **21. Providing assistance to team members is a sign that an individual does not have enough work to do** *

Mark only one oval.

- Strong disagree
- Disagree
- Neutral
- Agree
- Strongly agree

50. **22. Offering to help a fellow team member with his/her individual work tasks is an effective tool for improving team performance** *

Mark only one oval.

- Strong disagree
- Disagree
- Neutral
- Agree
- Strongly agree

51. **23. It is appropriate to continue to assert a patient safety concern until you are certain that it has been heard.** *

Mark only one oval.

- Strong disagree
- Disagree
- Neutral
- Agree
- Strongly agree

52. **24. Personal conflicts between team members do not affect patient safety** *

Mark only one oval.

- Strong disagree
- Disagree
- Neutral
- Agree
- Strongly agree

Communication

53. **25. Teams that do not communicate effectively significantly increase their risk of committing errors** *

Mark only one oval.

- Strong disagree
- Disagree
- Neutral
- Agree
- Strongly agree

54. **26. Poor communication is the most common cause of reported errors** *

Mark only one oval.

- Strong disagree
- Disagree
- Neutral
- Agree
- Strongly agree

55. **27. Adverse events may be reduced by maintaining an information exchange with patients and their families** *

Mark only one oval.

- Strong disagree
- Disagree
- Neutral
- Agree
- Strongly agree

56. **28. I prefer to work with team members who ask questions about information I provide** *

Mark only one oval.

- Strong disagree
- Disagree
- Neutral
- Agree
- Strongly agree

57. **29. It is important to have a standardized method for sharing information when handing off patients after endoscopy** *

Mark only one oval.

- Strong disagree
- Disagree
- Neutral
- Agree
- Strongly agree

58. **30. It is nearly impossible to train individuals how to be better communicators** *

Mark only one oval.

- Strong disagree
- Disagree
- Neutral
- Agree
- Strongly agree

59. **Please indicate any additional comments in the space below.**

Thanks for your participation.

Powered by
Google Forms
