# Supplementary material for: Simulation-Based Training of Non-Technical Skills in Colonoscopy: Protocol for a Randomized Controlled Trial
Source: JMIR Res Protoc. 2017 Aug 4;6(8):e153. doi: 10.2196/resprot.7690 (PMC5562936; doi:10.2196/resprot.7690)
Supplement: Multimedia Appendix 3 [file resprot_v6i8e153_app3.pdf]

Appendix II:
Endoscopic Non-technical Skills (E-NTS) Checklist for Non-technical Skills in Endoscopy

Non-technical Skill	Yes	No
Able to take focused patient history		
Reviews medications (i.e. anticoagulants) and allergies		
Identifies correct procedure and obtains consent		
Discusses with anesthetist/RN regarding sedation plan		
Situates patient in correct position		
Identifies issues/problems during procedure and verbalizes plan		
Addresses patient comfort (i.e. asks patient where pain is) and enacts plan		
Verbalizes/acknowledges relevant anatomy/landmarks		
Checks vital signs every few minutes		
Identifies issues/problems during procedure and verbalizes plan		
Updates team and patient regarding progress of procedure		
Introduces him/herself and team to patient		
Discusses procedure with patient and addresses concerns		
Asks team if they are ready to start		
Uses closed-loop communication when interacting with team		
Discusses results with patient post-procedure (i.e. complication, what was found, etc.)		
Respectful to patient and team		
