# Supplementary material for: Simulation-Based Training of Non-Technical Skills in Colonoscopy: Protocol for a Randomized Controlled Trial
Source: JMIR Res Protoc. 2017 Aug 4;6(8):e153. doi: 10.2196/resprot.7690 (PMC5562936; doi:10.2196/resprot.7690)
Supplement: Multimedia Appendix 4 [file resprot_v6i8e153_app4.pdf]

DOPS COLONOSCOPY ASSESSMENT FORM

Endoscopist Participant ID #: _______ Procedure Date (DD/MM/YYYY): _______________

Assessor: ________________________ Procedure # (circle one): 1 2

Please write the appropriate score from the scale below

Scale:
- **4** Highly skilled performance
- **3** Competent & safe throughout procedure, no uncorrected errors
- **2** Some standards not yet met, aspects to be improved, some errors uncorrected
- **1** Accepted standards not yet met, frequent errors uncorrected
- **N/A** Not applicable

CRITERIA	SCORE
**Assessment, consent, communication**	
• Obtains informed consent using a structured approach<br>o Satisfactory procedural information<br>o Risk and complications explained<br>o Co-morbidity<br>o Sedation<br>o Opportunity for questions	**N/A 1 2 3 4**
• Demonstrates respect for patient's views and dignity during the procedure	**N/A 1 2 3 4**
• Communicates clearly with patient, including outcome of procedure with appropriate management and follow up plan. Full endoscopy report.	**N/A 1 2 3 4**
**Safety and sedation**	
• Safe and secure IV access	**N/A 1 2 3 4**
• Gives appropriate dose of analgesia and sedation and ensures adequate oxygenation and monitoring of patient	**N/A 1 2 3 4**
• Demonstrates good communication with the nursing staff, including dosages and vital signs	**N/A 1 2 3 4**
**Endoscopic skills**	
o Checks endoscope function before intubation	**N/A 1 2 3 4**
o Performs PR	**N/A 1 2 3 4**
• Maintains luminal view / inserts in luminal direction	**N/A 1 2 3 4**
• Demonstrates awareness of patient's consciousness and pain during the procedure and takes appropriate action	**N/A 1 2 3 4**
o Uses torque steering and control knobs appropriately	**N/A 1 2 3 4**
o Uses distension, suction and lens washing appropriately	**N/A 1 2 3 4**
• Recognises and logically resolves loop formation	**N/A 1 2 3 4**
o Uses position change and abdominal pressure to aid luminal views	**N/A 1 2 3 4**
o Completes procedure in reasonable time	**N/A 1 2 3 4**
**Diagnostic and therapeutic ability**	
• Adequate mucosal visualisation	**N/A 1 2 3 4**
• Recognises caecal/desc. colon landmarks or incomplete examination	**N/A 1 2 3 4**
• Accurate identification and management of pathology	**N/A 1 2 3 4**
• Uses diathermy and therapeutic techniques appropriately and safely	**N/A 1 2 3 4**
• Recognises and manages complications appropriately	**N/A 1 2 3 4**
