# Supplementary material for: Simulation-Based Training of Non-Technical Skills in Colonoscopy: Protocol for a Randomized Controlled Trial
Source: JMIR Res Protoc. 2017 Aug 4;6(8):e153. doi: 10.2196/resprot.7690 (PMC5562936; doi:10.2196/resprot.7690)
Supplement: Multimedia Appendix 5 [file resprot_v6i8e153_app5.pdf]

M-OSANTS – NON-TECHNICAL SKILLS

SITUATIONAL AWARENESS: sample questions are listed below, give a global rating.

1 - Fail	2 - Poor	3 - Average	4 - Very good	5 - Excellent

Was the endoscopist able to remain aware of the patient's history (e.g. allergies, medications, etc.)?
Did the endoscopist review procedural details prior to procedure (e.g. confirms correct procedure)?
Did the endoscopist demonstrate procedural planning (e.g. identifies objectives for the procedure at the start)?
Did the endoscopist collect and use information during the procedure (e.g. change in vital signs)?
Did the endoscopist recognize the scope of practice (e.g. refrain from unfamiliar procedures/ interventions)?
Did the endoscopist anticipate potential problems during the procedure while proposing suitable solutions (e.g. proactively apply loop reduction strategies)?
Was the endoscopist mindful of procedure time?
Did the endoscopist ensure that patient outcomes are met (e.g. maintain patient comfort)?
Did the endoscopist anticipate needs of team members and of the patient (e.g. minimize patient anxiety)?

DECISION MAKING: sample questions are listed below, give a global rating.

1 - Fail	2 - Poor	3 - Average	4 - Very good	5 - Excellent

Was the endoscopist able to implement endoscopic and clinical knowledge when making a decision (e.g. choosing equipment appropriate to endoscopic appearance)?
Did the endoscopist identify issues and subsequently tailor a plan for resolution (e.g. application of loop reduction strategies)?
Did the endoscopist confidently create a plan and articulate details of the plan to the team)?
Did the endoscopist demonstrate understanding of the risks and benefits of an intervention/ maneuver (e.g. aware of bleeding risk due to polypectomy)?
Did the endoscopist account for relevant patient information (e.g. mindful of contraindications)?
Did the endoscopist appropriately delegate tasks to staff (e.g. requesting equipment from nurses)?
Did the endoscopist enact a subsequent option if initial action unsuccessful?
Did the endoscopist respond appropriately if the procedure extends out of the endoscopist's scope of practice (e.g. asking for assistance from senior staff)?

COMMUNICATION: sample questions are listed below, give a global rating.

1 - Fail	2 - Poor	3 - Average	4 - Very good	5 - Excellent

Was the endoscopist able to receive and respond to information from team members?
Did the endoscopist actively limit distractions in the room (e.g. restricts cell phone use)?
Did the endoscopist convey information using a closed-loop (e.g. confirms amount of sedation to be administered)?
Did the endoscopist speak with clarity, while providing details when appropriate (e.g. requesting snare with specific size)?
Did the endoscopist indicate a specific team member if there are multiple staff (e.g. addresses nurse by name)?
Did the endoscopist use language appropriate for the recipient (e.g. minimizes medical jargon for patients)?
Was the endoscopist aware of verbal tone and volume (e.g. speaks to staff in a respectful, collegial manner that can be heard)?
Did the endoscopist ensure that the recipient understands information (e.g. patient comprehends risks)?
Did the endoscopist relay findings to patient, including any adverse events (e.g. follow-up during aftercare)?

LEADERSHIP: sample questions are listed below, give a global rating.

1 - Fail	2 - Poor	3 - Average	4 - Very good	5 - Excellent

Was the endoscopist able to take responsibility for the process of the procedure (e.g. acknowledge mistakes)?
Did the endoscopist direct the flow of the team process, including an appropriate delegation of labour (e.g. requesting that nurses attend to patient discomfort)?
Did the endoscopist demonstrate confidence when leading the team, even under pressure (e.g. maintains composure during a bleed)?
Did the endoscopist lead the endoscopic pause?

PROFESSIONALISM: sample questions are listed below, give a global rating.				
**1 - Fail**	**2 - Poor**	**3 - Average**	**4 - Very good**	**5 - Excellent**

Did the endoscopist demonstrate a respectful and courteous attitude towards the patient and team members (e.g. introduces himself/herself to everyone in the room)?
Did the endoscopist acknowledge mistakes during procedure?
Did the endoscopist display empathy for the patient (e.g. responds to patient discomfort)?
Did he endoscopist advocate on behalf of the patient?
Did the endoscopist manage time appropriately (e.g. mindful of endoscopy unit time)?
Did the endoscopist ensure follow-up and address patient concerns within appropriate environment (e.g. follow-up within office or dedicated clinical area)?
Did the endoscopist refrain from inappropriate conversations (e.g. does not discuss other patients during a procedure)?
Did the endoscopist ensure that the procedure adheres to best-practice guidelines (e.g. record quality metrics)?

TEAMWORK: sample questions are listed below, give a global rating.				
**1 - Fail**	**2 - Poor**	**3 - Average**	**4 - Very good**	**5 - Excellent**

Was the endoscopist able to act effectively within the team of nurses, technicians, management, and other physicians?
Did the endoscopist demonstrate respect for all members of the team (e.g. speaks in a collegial, respectful tone)?
Was the endoscopist aware of the roles of all members of the endoscopic team?
Did the endoscopist display willingness to assist others, if appropriate (e.g. when transferring a patient)?
Did the endoscopist ask for advice from other team members?
Did the endoscopist take into account feedback from other team members (e.g. listens to suggestions for equipment)?
