# Supplementary material for: Simulation-Based Training of Non-Technical Skills in Colonoscopy: Protocol for a Randomized Controlled Trial
Source: JMIR Res Protoc. 2017 Aug 4;6(8):e153. doi: 10.2196/resprot.7690 (PMC5562936; doi:10.2196/resprot.7690)
Supplement: Multimedia Appendix 6 [file resprot_v6i8e153_app6.pdf]

GASTROINTESTINAL ENDOSCOPY COMPETENCY ASSESSMENT TOOL (GiECAT)

1) Using the scale provided, please rate the candidate's performance on the following global rating items:

Please note: A score of 4 should be assigned to those individuals who are *competent* to perform the tasks *independently*, *without* the need for *supervision* (i.e., do not take the candidate's level of training into account in assigning a score)

SCALE:

- **1** Unable to achieve tasks despite significant verbal and/or hands-on guidance
- **2** Achieves some of the tasks but requires significant verbal and/or hands-on guidance
- **3** Achieves most of the tasks independently, with minimal verbal and/or manual guidance
- **4** Competent for independent performance of all tasks
- **5** Highly skilled performance of all tasks

GLOBAL RATING ITEM	SCORE
**TECHNICAL SKILL**<br>*Demonstrates an ability to manipulate the endoscope using torque steering, angulation control knobs and advancement/withdrawal for effective navigation of the gastrointestinal tract.*	
**STRATEGIES FOR ENDOSCOPE ADVANCEMENT**<br>*Demonstrates an ability to use loop-reduction, insufflation, pull-back, suction, external pressure and patient position change to advance the endoscope independently, expediently and safely.*	
**VISUALIZATION OF MUCOSA**<br>*Demonstrates an ability to achieve a clear luminal view required for safe endoscope navigation and complete mucosal evaluation, including good visualization around corners and folds and appropriate use of mucosal cleaning techniques (e.g. lavage, suction).*	
**INDEPENDENT PROCEDURE COMPLETION (NEED FOR ASSISTANCE)**<br>*Demonstrates an ability to complete the endoscopic procedure expediently and safely without verbal and/or manual guidance.*	
**KNOWLEDGE OF PROCEDURE**<br>*Demonstrates general procedural knowledge including indications and contraindications, potential complications, endoscopy techniques, equipment maintenance and trouble-shooting.*	
**INTERPRETATION AND MANAGEMENT OF FINDINGS**<br>*Demonstrates an ability to accurately identify and interpret pathology and/or procedural complications and form an appropriate management plan.*	
**PATIENT SAFETY**<br>*Demonstrates an ability to perform the procedure in a manner that minimizes patient risk and assures optimal patient safety (e.g., atraumatic technique, minimal force, minimal red-out, recognition of personal and procedural limitations, safe sedation practices, and appropriate communication).*	

2) Please rate the candidate's performance on the following checklist items (check the appropriate box):

	ITEM	Not Done or Done Incorrectly	Done Correctly	N/A
PRE-PROCEDURE				
1.	Reviews relevant patient information (health records, relevant investigations) and obtains history as appropriate (indications, contraindications, medical history, medications, allergies).	☐	☐	☐
2.	Takes action in response to patient history and investigations where appropriate (e.g. prophylactic antibiotics, anesthetic risk factors, etc.).	☐	☐	☐
3.	Demonstrates a sound knowledge of the indications and contraindications to colonoscopy, its benefits and risks, potential alternative investigations and/or therapies, and an awareness of the sequelae of endoscopic or non-endoscopic management.	☐	☐	☐
4.	Explains to the patient and/or caregivers the perioperative process and procedure (likely outcome, time to recovery, benefits, potential risks/complications and rates), checks for understanding and addresses concerns and questions.	☐	☐	☐
PROCEDURE - TECHNICAL				
5.	Recognizes loop formation and avoids or reduces appropriately during the procedure (using pull-back, torque, external pressure, patient position change).	☐	☐	☐
6.	Uses rotation and/or torque appropriately.	☐	☐	☐
7.	Uses withdrawal (as an advancement strategy) appropriately.	☐	☐	☐
8.	Uses abdominal pressure and changes in patient position appropriately to aid scope advancement.	☐	☐	☐
9.	Advances to cecum (in an appropriate time)	☐	☐	☐
10.	Withdraws from cecum/terminal ileum to rectum in an appropriate time (> 6 min).	☐	☐	☐
11.	Withdraws endoscope in a controlled manner.	☐	☐	☐
12.	Performs therapeutic maneuvers (biopsy and/or polypectomy) independently, appropriately and safely.	☐	☐	☐
PROCEDURE - COGNITIVE				
13.	Demonstrates recognition of anatomical landmarks (rectum, flexures, ileo-cecal valve, appendiceal orifice, etc.) and/or incomplete examination.	☐	☐	☐
14.	Demonstrates recognition of pathological and anatomical abnormalities.	☐	☐	☐
15.	Describes findings accurately, interprets abnormalities in the context of the patient and selects the appropriate strategy/technique to deal with them.	☐	☐	☐
PROCEDURE - NON-TECHNICAL				
16.	Administers sedation appropriately (type, dose), monitors the patient's vitals and comfort level throughout the procedure and responds appropriately AND/OR demonstrates appropriate interaction with the anesthetist to ensure appropriate sedation and monitoring throughout the procedure.	☐	☐	☐
17.	Demonstrates appropriate interaction and communication with the procedure nurses and/or assistants throughout the procedure.	☐	☐	☐
POST-PROCEDURE				
18.	Educates the patient and/or caregiver about the colonoscopic findings (explanation, significance) and follow-up plan, and provides advice regarding potential post-procedure complications, recommended course of action, etc.	☐	☐	☐
19.	Appropriate and timely documentation of procedure (written/dictated/EMR)	☐	☐	☐

3) Please indicate if a lapse in professionalism occurred:

- ☐ No
- ☐ Minor lapse (inadvertent and/or did not cause any substantial harm)
- ☐ Major lapse (evidence of full knowledge that this action was not right and/or the lapse does cause harm)

4) OVERALL ASSESSMENT

a) Overall, the endoscopist's ***hands-on (technical) skills*** are:

- ☐ Novice
- ☐ Beginner
- ☐ Intermediate
- ☐ Competent
- ☐ Expert

b) Overall, the endoscopist's ***knowledge*** about colonoscopy is:

- ☐ Novice
- ☐ Beginner
- ☐ Intermediate
- ☐ Competent
- ☐ Expert

c) Overall, the endoscopist's ***higher-order skills*** (e.g. clinical reasoning, judgment, interpretation of abnormalities, situational awareness, communication, ethical integrity) are:

- ☐ Novice
- ☐ Beginner
- ☐ Intermediate
- ☐ Competent
- ☐ Expert

5) ASSESSOR GLOBAL ASSESSMENT

In order to aid in the validation process, please provide your expert global assessment of the edoscopist's skill level *independent of the above checklist and global ratings.*

- ☐ Requires significant guidance with all aspects of performing colonoscopy
- ☐ Able to perform colonoscopy under supervision, but requires guidance with many aspects of the procedure
- ☐ Able to perform colonoscopy independently, under supervision, with minimal guidance
- ☐ Competent to perform colonoscopy independently, safely and expediently without the need for supervision
- ☐ Highly skilled advanced ability to perform colonoscopy independently, with optimal efficiency and safety

OTHER MEASURES

6) Overall comfort of the patient:

No discomfort	Minimal discomfort	Mild discomfort	Moderate discomfort	Severe discomfort
1	2	3	4	5

7) Case difficulty:

Extremely easy	Fairly easy	Average	Fairly difficulty	Extremely difficulty
1	2	3	4	5

8) BOWEL PREPARATION

a) The efficacy of the bowel preparation was:

Inadequate	Poor	Fair	Good	Excellent
Solid stool obscuring mucosal detail and contour despite aggressive washing and suctioning.	*Presence of stool obscuring mucosal detail and contour. However, with suctioning and washing a reasonable view is obtained*	*Turbid fluid or stool residue obscuring mucosal detail. However, mucosal detail becomes visible with suctioning. Washing not necessary.*	*Some turbid fluid or stool residue but mucosal detail still visible. Washing and suctioning not necessary.*	*Mucosal detail clearly visible. If fluid present, it is clear. Almost no stool residue*
1	2	3	4	5

b) Ottawa Bowel Preparation Scale:

i) Fluid in the whole colon:

- ☐ Small
- ☐ Moderate
- ☐ Large
- ☐ N/A

ii) Please rate the cleanliness of the:

**SECTION OF COLON**	Inadequate<br>*Stool obscures despite major wash/suction*<br>1	Poor<br>*Stool obscures, OK view with suction/wash*<br>2	Fair<br>*Fluid/stool obscuring mucosa, seen with suction, wash not needed*<br>3	Good<br>*Turbid fluid/stool but mucosa visible, wash/suction not needed*<br>4	Excellent<br>*Mucosal detail visible, no/clear fluid, almost no stool*<br>5
**RECTO-SIGMOID**					
**MID COLON**					
**RIGHT COLON**					

9) How easy was it for you to rate the endoscopist's performance using this tool?

Extremely easy	Fairly easy	Average	Fairly difficulty	Extremely difficulty
1	2	3	4	5
