# Supplementary material for: Simulation-Based Training of Non-Technical Skills in Colonoscopy: Protocol for a Randomized Controlled Trial
Source: JMIR Res Protoc. 2017 Aug 4;6(8):e153. doi: 10.2196/resprot.7690 (PMC5562936; doi:10.2196/resprot.7690)
Supplement: Multimedia Appendix 7 [file resprot_v6i8e153_app7.pdf]

Endoscopist Participant ID #: __________ Date (DD/MM/YYYY): ____________ Start Time: __________

End Time: __________

Assessor: _________________ Clinical Procedure (circle one): 1 2 3

NURSE ASSESSED PATIENT COMFORT SCORE (NAPCOMS)

Domain	Item	0	1	2	3	Score
Pain	1- Intensity	None or minimal	Mild	Moderate	Severe	
	2- Frequency	None	Few (1 or 2 episodes)	Several times (3-4 episodes)	Frequent (>4 episodes)	
	3- Duration	None	Short duration (episode <30 seconds)	Moderate duration (30 sec-1 minute)	Long duration (episode lasts >1 min)	
	Total Pain Score (Intensity + Frequency + Duration)					
Sedation	Level of consciousness*	Alert	Sleepy but initiates conversation	Responds only when asked or stimulated	Unresponsive or only responds with pronounced simulation	
Global	Tolerability*	Very well tolerated	Reasonably well tolerated	Just tolerated	Poorly tolerated	

*Note: level of consciousness and tolerability are not used in overall score
