# Supplementary material for: Simulation-Based Training of Non-Technical Skills in Colonoscopy: Protocol for a Randomized Controlled Trial
Source: JMIR Res Protoc. 2017 Aug 4;6(8):e153. doi: 10.2196/resprot.7690 (PMC5562936; doi:10.2196/resprot.7690)
Supplement: Multimedia Appendix 8 [file resprot_v6i8e153_app8.pdf]

THE GENERAL SELF-EFFICACY SCALE

Please rate the following items based on a 4-rank scale.

1= Not at all true 2= Hardly true 3= Moderately true 4= Exactly true

ITEM	RATING
I can always manage to solve difficult problems if I try hard enough.	
If someone opposes me, I can find the means and ways to get what I want.	
It is easy for me to stick to my aims and accomplish my goals.	
I am confident that I could deal efficiently with unexpected events.	
Thanks to my resourcefulness, I know how to handle unforeseen situations.	
I can solve most problems if I invest the necessary effort.	
I can remain calm when facing difficulties because I can rely on my coping abilities.	
When I am confronted with a problem, I can usually find several solutions.	
If I am in trouble, I can usually think of a solution.	
I can usually handle whatever comes my way.	
