# Supplementary material for: Simulation-Based Training of Non-Technical Skills in Colonoscopy: Protocol for a Randomized Controlled Trial
Source: JMIR Res Protoc. 2017 Aug 4;6(8):e153. doi: 10.2196/resprot.7690 (PMC5562936; doi:10.2196/resprot.7690)
Supplement: Multimedia Appendix 9 [file resprot_v6i8e153_app9.pdf]

INTEGRATED SCENARIO GLOBAL RATING FORM

Please rate the endoscopist's performance on the following items:

		Below expectations for starting clinical duty		Borderline for starting clinical duty	Meets expectations for starting clinical duty	Above expectations for starting clinical duty		Unable to comment
1	Introduction/establish rapport	1	2	3	4	5	6	7
2	Explanation of intervention including patient's consent to proceed	1	2	3	4	5	6	7
3	Assessment of patient's and parent's needs before procedure	1	2	3	4	5	6	7
4	Preparation for procedure	1	2	3	4	5	6	7
5	Technical performance of procedure	1	2	3	4	5	6	7
6	Maintenance of asepsis	1	2	3	4	5	6	7
7	Awareness of patient's and parent's needs during procedure	1	2	3	4	5	6	7
8	Closure of the procedure including explanation of follow-up care	1	2	3	4	5	6	7
9	Clinical safety	1	2	3	4	5	6	7
10	Professionalism	1	2	3	4	5	6	7
11	Overall ability to perform the procedure (including technical and professional skills)	1	2	3	4	5	6	7

How would you rate the endoscopist's overall performance (circle one):

Incompetent Borderline Competent
