# Supplementary material for: Simulation-Based Training of Non-Technical Skills in Colonoscopy: Protocol for a Randomized Controlled Trial
Source: JMIR Res Protoc. 2017 Aug 4;6(8):e153. doi: 10.2196/resprot.7690 (PMC5562936; doi:10.2196/resprot.7690)
Supplement: Multimedia Appendix 10 [file resprot_v6i8e153_app10.pdf]

INTEGRATED SCENARIO COMMUNICATION RATING FORM

Circle the rating which best reflects your judgement of the endoscopist's overall performance with regard to communication:

OVERALL ASSESSMENT OF THE KNOWLEDGE AND SKILLS DEMONSTRATED IN THE INTERVIEW		
1 **2**	**3 4 5**	**6 7**
Responds **inappropriately** and **ineffectively** to the task indicating a **lack of knowledge** *and/or* **undeveloped interpersonal and interviewing skills.**	Responds **effectively** to **some** components of the task indicating **an adequate knowledge base** *and* **some development of interpersonal and interviewing skills**	Responds **precisely and perceptively** the task, **consistently integrating all components**.

Circle the rating which best reflects your judgement of the endoscopist's performance in the following categories of communication:

RESPONSE TO PATIENT'S AND PARENT'S FEELINGS AND NEEDS (EMPATHY)		
**1 2**	**3**	**4 5**
Does **not** respond to obvious patient cues *and/or* responds **inappropriately**	Responds to patient's needs and cues, but **not always effectively.**	Responds **consistently** in a **perceptive** and **genuine** manner to the patient's needs and cues.
**DEGREE OF COHERENCE IN THE INTERVIEW**		
**1 2**	**3**	**4 5**
**No recognizable plan** to the interaction, the plan does **not** demonstrate cohesion, *or* the **patient** must determine direction of the interview	Organizational approach is **formulaic** and **minimally flexible** *and/or* control of the interview is **inconsistent**	**Superior organization**, demonstrating **command** of cohesive devises, **flexibility,** and **consistent control** of the interview
**VERBAL EXPRESSION**		
**1 2**	**3**	**4 5**
Communicates in manner that **interferes with** *and/or* **prevents understanding** by patient	Exhibits **sufficient control** of expression to be **understood** by an active listener (patient)	Exhibits **command** of expression (fluency, grammar, vocabulary, tone, volume and modulation of voice, rate of speech, pronunciation)
**NON-VERBAL EXPRESSION**		
**1 2**	**3**	**4 5**
**Fails to engage, frustrates** *and/or* **antagonizes** the patient	Exhibits **enough control** of non-verbal expression to **engage** a patient willing to overlook deficiencies such as passivity, self-consciousness, or inappropriate aggressiveness	Exhibits **finesse and command** of non-verbal expression (eye contact, gesture, posture, use of silence, etc.)
